# The results of surgical treatment of preschool and primary school age children with congenital deformation of the spine in isolated hemivertebra: Comparative analysis

**DOI:** 10.3389/fped.2022.960209

**Published:** 2022-09-09

**Authors:** Nurbek Nadirov, Sergey Vissarionov, Alexandra Filippova, Dmitriy Kokushin, Vitaliy Sazonov

**Affiliations:** ^1^Mother and Child Health Center, Department of Orthopedics, University Medical Center, Nur-Sultan, Kazakhstan; ^2^G. Turner National Medical Research Center for Children’s Orthopedics and Trauma Surgery, Ministry of Health of the Russian Federation, Saint Petersburg, Russia; ^3^Department of Biomedical Sciences, School of Medicine, Nazarbayev University, Nur-Sultan, Kazakhstan

**Keywords:** congenital kyphoscoliosis, isolated hemivertebra, spinal deformity, comparative analysis, dysplastic course, pediatric surgery

## Abstract

**Introduction:**

Despite a detailed study of the natural development of congenital spinal deformity in an isolated hemivertebra and the methods of surgical correction of this pathology, some issues remain unresolved. The age at which the surgical correction of congenital spinal deformity should be performed is a controversial issue among specialists dealing with this problem. The aim of the work was to conduct a comparative analysis of the results of spinal deformity correction in children with congenital kyphoscoliosis with an isolated hemivertebra of preschool and primary school age.

**Materials and methods:**

The study involved 26 patients aged from 1 year 9 months to 9 years 6 months (10 girls and 16 boys) with congenital kyphoscoliosis caused by an isolated hemivertebra. The patients underwent surgical interventions of partial or complete resection of the hemivertebra with adjacent intervertebral discs from the dorsal or combined approach, correction, and stabilization of congenital deformity of the spine with a posterior multi-support metal structure. All the patients were divided into two groups by age: the first group—children under 4 years old (14 children), and the second group—children of 6 years and older (12 children).

**Results:**

Metal fixation during surgical treatment in children of primary school and preschool ages was carried out in the majority of cases in a polysegmental manner. Regarding the approach for surgical treatment, it can be noted that in the 2nd group of patients, preference was more often given to the dorsal surgical approach. The duration of the surgical intervention and the amount of blood loss between different age groups did not have statistically significant differences. In the group of children of preschool age, in three cases, the destabilization of the metal structure was noted in the early postoperative period when control radiographs were performed after surgical treatment. In the group of older children, after surgical treatment, the spinal dysplastic deformity above or below the zone of metal fixation was detected in three cases.

**Conclusion:**

The effectiveness of surgical treatment of congenital deformity was significantly higher in children of the younger age group compared to school-age patients.

## Introduction

Congenital kyphoscoliosis with an isolated abnormally developed vertebra is one of the most common malformations of the spinal column. The nature of the course of congenital spinal deformity in this variant of the anomaly has been studied in sufficient detail and covered in the literature (1). Methods and approaches to the surgical treatment of the patients with congenital scoliosis associated with an isolated disorder of vertebral formation are described extensively, methods of combined and dorsal approaches to the body of the hemivertebra are developed, and variants of instrumental fixation are described (mono- or bilateral fixation, poly or monosegmental metal fixation, the use of hook, hybrid or transpedicular systems) for deformity correction (2). However, despite the available research on the natural course of congenital scoliosis with an abnormally developed vertebra and the tactics of treating pediatric patients with congenital curvatures, some issues remain unresolved. One of the most discussed issues in the treatment of children with congenital deformities of the spinal column is the optimal age of the patients at which it is necessary to perform the surgery. In the available literature, there is not much data on a targeted comparative analysis of the results of surgical treatment of congenital spinal deformities in children of different age groups. Several authors recommend performing the surgical treatment of children with congenital spinal deformities as early as possible in order to achieve the optimal result of curvature correction (3–5). However, it is also reported that due to the immaturity of the bone tissue, the narrow dimensions of the base of the vertebral arches, and the low strength of bone structures in patients of early age, such complications in the postoperative period as the destabilization of the metal structure are more often observed (6, 7). Other researchers argue that an operation aimed at correcting a congenital spinal deformity is possible even at the patient’s school age. In this age period, from their point of view, it is also possible to achieve the optimal result of the correction of congenital curvature of the spinal column (8). Thus, the question of the optimal age for surgical intervention in children with congenital spinal deformities remains relevant and has not been fully resolved.

The aim of this work was to conduct a comparative analysis of the results of surgical treatment of children of preschool and primary school age with congenital scoliosis.

## Materials and methods

The article analyzes the results of the surgical treatment of 26 children with congenital scoliosis with an isolated vertebral formation disorder in the lower thoracic and lumbar spine. There were 16 boys and 10 girls among the patients. The average age of patients was 4 years 5 months (from 1 year 9 months to 9 years 6 months).

All the patients underwent surgical interventions at the National Research Center for Pediatric Traumatology and Orthopedics named after G.I. Turner in the amount of partial or complete resection of the hemivertebra with adjacent intervertebral discs from the dorsal or combined approach, correction and stabilization of congenital deformity of the spine with a posterior multi-support metal structure, and dorsal fusion. The same surgical team operated all the patients. Patients underwent a standard preoperative examination, including clinical and laboratory examinations, vertebral spondylograms in frontal and lateral projections, MSCT of the thoracic and lumbar spine, and magnetic resonance imaging. After surgical treatment, all children underwent an X-ray of the spine in frontal and lateral projections and MSCT of the metal fixation zone. All the children after the surgical treatment received antibiotic prophylaxis, symptomatic treatment, were verticalized on days 3–5 after surgery, and on days 7–10 all the children were prescribed to wear a hard corset.

The inclusion criteria for the study were: congenital kyphoscoliosis with an isolated hemivertebra located in the lower thoracic or lumbar spine; the age of patients from 1 to 10 years at the time of surgical treatment; surgical intervention in the amount of partial or complete resection of a hemivertebra with an adjacent disc apparatus from a dorsal or combined approach, correction and stabilization of congenital spinal deformity with a posterior multi-support metal structure, and dorsal fusion. Exclusion criteria: spinal deformity caused by other variants of anomalies of its development; concomitant pathology of the spinal cord; and the refusal of the patient or his representative from surgical treatment and participation in the study.

The mean follow-up period for patients was 2 years 11 months (from 2 years 4 months to 4 years 1 month). All the children in the process of observation were assessed by the clinical status and x-ray picture every 6 months. Radiographs were used to assess local scoliotic and kyphotic deformities of the spine with a congenital anomaly of the spine according to Cobb before the surgery, the amount of correction after surgical treatment, the stability of the metal structure, and the presence of a dysplastic deformity outside the area of metal fixation during observation.

The assessment of the surgical treatment of children of different age groups was carried out by following parameters: the surgical approach (dorsal or combined), the length of metal fixation, the duration of the surgical intervention, the volume of intraoperative blood loss, the amount of correction of the scoliotic and kyphotic components of congenital deformity, the percentage of destabilization of metal structures, and the presence of dysplastic course of congenital deformity of the spine (appearance of a secondary deformity of the spine with a torsion component above or below the area of surgical intervention during the growth and development of the child).

Statistical data analysis was performed using online medical statistics calculators. To determine the statistical significance of differences in paired measurements, the Mann–Whitney *U* test was used (for small samples), the results were considered significant at *p* < 0.05.

## Results

All the patients were divided into two groups depending on their age: the first group—children under 4 years old (from 1 year 9 months to 3 years 11 months)—14 children, and the second group—children from 6 years and older (from 6 years 1 month up to 9 years 6 months)—12 children (Table 1). Among the patients, in 18 children the hemivertebra was localized in the lumbar region (in 3 patients—L1 hemivertebra, in 9—L2 hemivertebra, in 5—L3 hemivertebra, and in 1 child—L4) and in 8 patients—in the lower thoracic region (in 2 children—Th9 hemivertebra, 4—Th10, and 2—Th11). Right-sided hemivertebrae occurred in 17 children (65% of cases), and in 9 children—left-sided (35% of cases). The value of the scoliotic component of local congenital deformity according to Cobb varied from 26^°^ to 40^°^, the value of the kyphotic component—from 8^°^ to 27^°^.

**TABLE 1:** Characteristics of the study groups by age, gender, and localization of the spinal malformation.

		Gender			Localization	
	Number of patients	Female	Male	Age mean in months (min; max)	Low-thoracic hemivertebra	Lumbar hemivertebra
First group	14	4	10	35 (21; 47)	4	10
Second group	12	8	4	85 (73; 110)	4	8

All the patients underwent surgical intervention aimed at extirpation or partial/complete resection of the hemivertebra, correction, and stabilization of congenital spinal deformity with a multi-support metal structure, for which 6 patients underwent the surgical treatment through the dorsal approach and 20 patients—through the combined approach. In all children, the correction and stabilization of congenital spinal deformity were performed with a bilateral metal construct with various layout options (transpedicular or hybrid metal fixation), in 6 patients—monosegmental fixation, and in 20 children—polysegmental fixation (Table 2).

**TABLE 2:** Characteristics of indicators of spinal deformity and features of surgical treatment.

	Local deformation in degrees by Cobb		Surgical approach		Length of metal construct	
	Scoliosis (M ± m), p = 0.004	Kyphosis (M ± m), p = 0.003	Dorsal	Combined	Monosegmental	Polysegmental
First group	30 **±** 2.8	16 + 1.9	2	12	5	9
Second group	29 + 2.9	10 + 2.3	4	8	1	11

As can be seen from Table 2, the magnitude of spinal deformity in children was comparable in both groups. However, if we take into account the age of the patients, it can be noted that the spinal deformity in patients of the first group (in children under 4 years old) was visually more pronounced than in children of the second group, which is because of the smaller volume of soft tissues in the area of congenital anomalies in young children. Most likely, the earlier appointment with the doctor depended on this fact. Metal fixation during surgical treatment in older children was carried out in the majority of cases (92% of cases) in a polysegmental manner (from 3 to 6 vertebrae). This length of fixation is related to the fact that in patients of the second group, a greater number of vertebrae were included in the main curve of the curvature compared with the comparison group, and this required the impact of a larger lever in order to achieve correction of the congenital deformity. Regarding the choice of access for surgical treatment, it can also be noted that in the second group of patients, the dorsal surgical approach was more often preferred (in 33% of cases compared to 14% in the 1st group) due to the reduction in the duration of the surgical intervention (Table 3). To be able to compare data on the blood loss between children of different age groups, due to the difference in the volume of circulating blood, the volume of blood loss was estimated as a relative blood loss in percentage of the circulating blood volume.

**TABLE 3:** Characteristics of surgical treatment parameters depending on surgical access in children of different age groups.

	Surgery length in minutes, mean (min; max)*		Relative (absolute, ml) blood loss (% of circulation blood volume), mean**		Correction: scoliosis; kyphosis (%), mean***	
	Dorsal approach	Combined approach	Dorsal approach	Combined approach	Dorsal approach	Combined approach
First group	215 (180; 230)	230 (205; 310)	19.5 (240 ml)	17.8 (225 ml)	93; 97	98; 97
Second group	220 (195; 250)	270 (245; 315)	20.1 (319 ml)	14.3 (280 ml)	88; 92	92; 93

*p = 0.09 for 1st and 2nd groups by operation duration. **p = 0.2 for 1st and 2nd groups by relative blood loss. ***p < 0.05 by correction amount.

**FIGURE 1:**
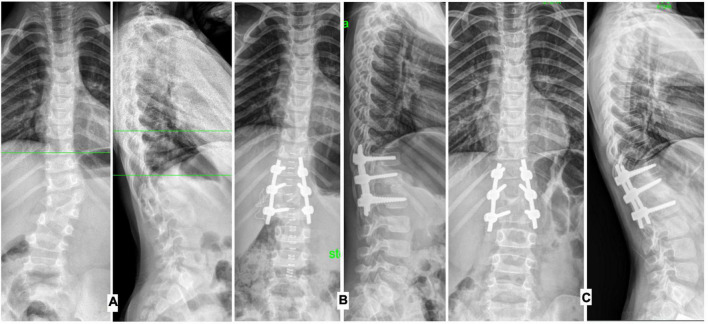
Radiographs of the spine in the frontal and lateral projections, operated at the age of 3 years 6 months: **(A)** before surgical treatment, **(B)** on the 6th day after surgical treatment, **(C)** 3 years after correction of congenital spinal deformity.

The duration of the surgical intervention and the amount of blood loss between different age groups did not have a statistically significant difference, both with the dorsal approach and with the combined approach, which indicates that the age of the child does not affect these indicators. The amount of correction in the group of children of preschool age was statistically greater compared to children of primary school age, both with the dorsal approach and with the combined one. In preschool children, the average correction was 93% for the scoliotic component (locally), 97% for the kyphotic component (locally) with the dorsal approach (Figure 1), 98 and 97%, respectively, with the combined approach. At primary school age, the percentage of deformity correction with dorsal access was scoliosis—88% and kyphosis—92%, with combined, respectively: 90 and 93%.

In addition to the main characteristics of surgical treatment and assessment of deformity correction, we analyzed the frequency of destabilization of metal structures in the study groups, and evaluated the long-term results of surgical treatment, i.e., formation of spinal deformity of a dysplastic nature outside the zone of metal fixation (Table 4).

**TABLE 4:** Short-term and long-term results of surgical treatment of congenital spinal deformity.

	Destabilization of metal construct, n (%)	Dysplastic changes, n (%)
First group	3 (21.4%)	1 (7.1%)
Second group	0	3 (25%)

In the group of preschool children, in three cases (21.4%), the destabilization of the metal structures was noted in the early postoperative period when control radiographs were done after surgical treatment (on days 7–10 after surgery). These were young children, who at the time of surgical treatment were from 1 year 11 months to 2 years 1 month. The destabilization of the hardware was associated with the small size of the bases of the arches of the vertebral bodies and the high stress on the bone structures of the latter, which occurred with the significant amount of correction of congenital deformity during the operation. All the children required surgery to restore the stability of the metal structure, and prolongation of metal fixation by one vertebra. In two cases, the reoperation was performed up to three weeks after surgical treatment, and in one case, 2 months after the first operation.

**FIGURE 2:**
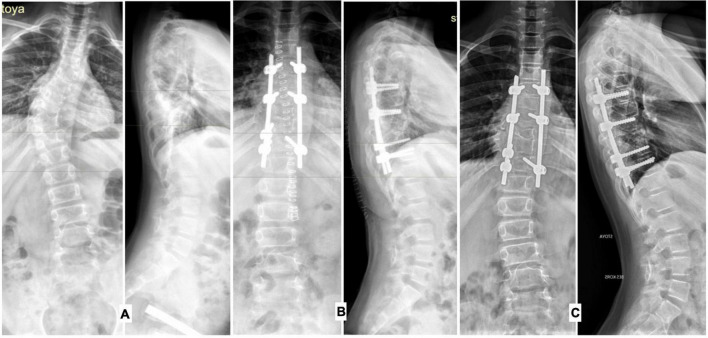
Radiographs of the spine in frontal and lateral projections, patient operated at the age of 9 years 6 months: **(A)** before surgical treatment, **(B)** on the 7th day after surgical treatment, **(C)** 1.5 years after correction of congenital deformity spine (dysplastic deformity of the spine below the zone of metal fixation).

The development of dysplastic spinal deformity in the observed patients outside the zone of metal fixation was noted 1.5–2 years after the surgical correction of congenital kyphoscoliosis with an isolated malformation of the vertebrae. The dysplastic course of congenital spinal deformity in the late period after surgical treatment was noted in 1 patient (the child was operated on at the age of 3 years 6 months, and the child’s parents refused to use a brace in the postoperative period) in the group of preschool children. In the group of older children, spinal deformity of dysplastic origin above and/or below the metal fixation zone was detected in three (25%) patients (Figure 2).

## Discussion

Surgical treatment of congenital deformities is a fairly well-studied issue; despite this, the tactical issues in choosing the scope and access of surgical intervention, and the age of the patient remain open. Indications for surgical treatment of congenital spinal deformity adopted at the Department of Spinal Pathology and Neurosurgery of the G. Turner are: local scoliotic deformity of more than 30°, local kyphosis of more than 8° for the lumbar region, and more than 18° for the thoracic region (9).

Choosing the previously named indicators for evaluating the results of surgical treatment, we compared groups of children of different ages with each other.

Thus, the duration of the surgical intervention and the amount of blood loss between different age groups did not have statistically significant differences, both with the dorsal approach and with the combined approach, which suggests that the age of the child factor does not affect these indicators. This fact is noted in the works by a number of researchers (10). The extent of metal fixation depended on the age of the child at the time of surgical treatment and access to surgical intervention. In older children, polysegmental fixation was performed in 92% of cases, in contrast to the small children—64%. At the same time, in the children of the younger age group, combined access was more often used (in 85.7% of cases), in contrast to the older children (66.6%).

The combined approach in the older age group took a longer time than in patients of an earlier age, which was the reason to choose the dorsal approach for surgical intervention in the older group of patients. The results of the surgical treatment in terms of deformity correction in young children were better than in patients of the older age group. From our point of view, this is explained by the fact that with the combined approach, it was possible to perform a complete and thorough mobilization of the spinal motion segment, and therefore, a “smaller lever” of action was required to correct spinal deformity.

The amount of deformity correction in the postoperative period in the group of preschool children was statistically greater compared to children of primary school age, which confirms the opinion of experts that surgical treatment of congenital spinal deformity at a young age gives better results in terms of the amount of correction of kyphoscoliotic curvature than in older children (11).

When evaluating the results of treatment in the postoperative period, it was noted that in the first group of patients, cases of destabilization of the metal structures were more often observed in the early postoperative period. In our opinion, this was due to the small size of the bases of the arches of the vertebral bodies, less durable bone structures, high stress during the correction of congenital deformity, and uncontrolled mobility of the child in the postoperative period, which increased the load on the implant bone. Prevention of these complications—early orthotics to exclude an increased range of motion of the spine in the area of metal fixation.

The occurrence of a dysplastic arch outside the zone of metal fixation was more often noted in children of the older age group, which was due to the fact that the long-term existence of spinal deformity formed a certain fixed pattern of trunk balance, which was changed as a result of deformity correction, after which the patients could not adapt to a new position in space. The manifestation of this was the formation of new arcs of curvature outside the area of surgical intervention. The objectives of this study did not include the assessment of the state of newly formed deformity arches outside the area of surgical intervention and their subsequent correction in the period of child growth. This situation indicates that surgical treatment, according to this criterion, at a younger age gives better long-term results compared to the older age group of children.

## Conclusion

A comparative analysis of the surgical treatment of spinal deformity in children of preschool and primary school age with congenital kyphoscoliosis with an isolated hemivertebra made it possible to draw the following conclusions.

The age of the child does not significantly affect the duration of the surgical intervention and the amount of intraoperative blood loss.

The amount of deformity correction in children of the younger age group was statistically greater than in the children of primary school age, both with the dorsal approach and with the combined one. Achieving a full-fledged correction in children of preschool age can often be achieved with a shorter metal fixation compared with older patients.

The percentage of the destabilization of metal structures in young children is higher, which requires repeated operations with an increase in the number of fixed vertebrae but does not lead to loss of correction of congenital spinal deformity. The spinal deformity (dysplastic course) outside the zone of metal fixation in school-age children develops more often than in patients of the younger age group. However, there is still some point of discussion here about the results, as it cannot be sufficiently clear whether the best result is mainly due to the younger age or to the type of approach most used in the lower age group.

## Data availability statement

The raw data supporting the conclusions of this article will be made available by the authors, without undue reservation.

## Ethics statement

The studies involving human participants were reviewed and approved by the University Medical Center. Written informed consent to participate in this study was provided by the participants’ legal guardian/next of kin.

## Author contributions

NN designed the study, collected the clinical data, and wrote the manuscript. SV contributed to the literature search, collecting of data, and drafting of the manuscript. AF and DK contributed to the collecting of data. VS contributed to writing the draft and critically revised the manuscript. All authors reviewed the draft, modified it accordingly, and approved the final version.
